# Portevin-Le Châtelier Effect in a Powder Metallurgy Co-Ni-Based Superalloy

**DOI:** 10.3390/ma15082796

**Published:** 2022-04-11

**Authors:** Chao Li, Jianwei Teng, Biaobiao Yang, Xianjue Ye, Lan Huang, Yong Liu, Yunping Li

**Affiliations:** 1 State Key Laboratory for Powder Metallurgy, Central South University, Changsha 410083, China; lichao1997@csu.edu.cn (C.L.); tengjianwei@csu.edu.cn (J.T.); biaobiaoyang@csu.edu.cn (B.Y.); yexianjue@csu.edu.cn (X.Y.); lhuang@csu.edu.cn (L.H.); yonliu@csu.edu.cn (Y.L.); 2 IMDEA Materials Institute, C/Eric Kandel 2, Getafe, 28906 Madrid, Spain; 3 Department of Materials Science, Polytechnic University of Madrid/Universidad Politécnica de Madrid, E.T.S. de Ingenieros de Caminos, 28040 Madrid, Spain

**Keywords:** Co-Ni-based superalloy, dynamic strain aging, Portevin-Le Châtelier (PLC) effect, strain rate sensitivity

## Abstract

The Portevin-Le Châtelier (PLC) effect in a powder metallurgy (PM) Co-Ni-based superalloy was systematically investigated via the tensile tests at temperatures ranging from 200 to 600 °C and strain rates at 1.0 × 10^−4^ to 1.0 × 10^−2^. Both normal and inverse PLC effects were observed in the PLC regime, and the former appeared in the A and B types at a low temperature, whilst the latter appeared in the C type at an elevated temperature. Both positive and negative strain rate sensitivities (SRS) were shown in PLC regime, and SRS should be derived from same types of serrations. Based on the calculated activation energy, the substitutional atom Mo is considered to take primary responsibility for the PLC effect in present alloy.

## 1. Introduction

Superalloys are widely used as hot-end parts due to their excellent combination of outstanding oxidation resistance, high strength, and superior creep resistance at elevated temperatures [1,2,3,4]. The Portevin-Le Châtelier (PLC) effect of superalloys that appears during servicing has attracted extensive attention due to its close relationship with the mechanical properties of alloy [5,6,7,8]. The PLC effect, depicting serrated flows in the stress–strain curves of alloys, is generally associated with the non-uniform plastic deformation behavior of the alloy within a certain regime of temperature and strain rate [9,10]. In the perspective of atomic scale, the PLC effect stems from the elastic interactions between mobile dislocations and diffusing atoms during deformation, which is also called dynamic strain aging (DSA) [11,12]. In plastic deformation, moving dislocations may be arrested transitorily by obstacles such as forest dislocations. Then, the solute atoms diffuse to the moving dislocations due to the lower activation energy for dislocation core diffusion compared to bulk diffusion [13]. The diffusion-induced solute atoms’ segregation behavior exerts a drag force on the moving dislocations. In the process, if the aging time $t_{a}$ (defined as the time for atoms to diffuse to arrest the dislocations) is less than the waiting time $t_{w}$ (defined as the time for moving dislocations to overcome the obstacles), the PLC effect or serrated flow may take place.

Many efforts have been devoted to understanding the underlying mechanism of the PLC effect. Increasing the temperature or decreasing the strain rate can promote the appearance of the PLC effect [7,14,15]. However, due to the complex composition of superalloys, a generally accepted viewpoint regarding which atom should be responsible for the PLC effect is still not clear. Nakada et al. [16] supported the significant role of interstitial C atoms on the PLC effect in Ni-based alloys. On the Contrary, Gopinath and Han [17,18] suggested that substitutional alloy atoms such as Cr, Co, Ti, Al, Mo, and W rather than interstitial atoms (C and B) should be responsible for the PLC effect in Udimet720Li. In addition, Hale and Chatterjee [6,19] hold that interstitial C atoms and substitutional atoms account for the PLC effect for low and elevated temperatures, respectively. Thorough investigation is still needed as to which alloying element is predominant the PLC effect.

Apart from the inconsistency regarding the correlation between the alloying element and the PLC effect, the relationship between macroscopic mechanical properties and the PLC effect in superalloys has also intrigued researchers for many years. One typical controversial point is the evolution of elongation with the presence of the PLC effect. The weakened role by the PLC effect was reported by Roy et al. [20], Gopinath et al. [17], and Tian et al. [21] on the elongation of austenitic Alloy C-276, Ni-base superalloy 720Li, and Ni-Co-based superalloy, respectively. In contrast, from the recent study by Pu et al. [14], the elongation of superalloy UNS N10276 was determined to abnormally increase when temperature was higher than 700 °C during the PLC effect regime. Their work also revealed disparate influence of the PLC effect on yield strength and ultimate tensile strength in comparison with the investigations by Gopinath et al. [17] and Tian et al. [21]. These contradictory reports demonstrate no consensus regarding the relationship between the PLC effect and macroscopic mechanical properties has been reached to date, unfortunately.

To tackle these problems, yield strength, ultimate tensile strength, as well as elongation in the PLC effect regime (temperatures: 200 to 600 °C, strain rates: 1.0 × 10^−2^, 1.0 × 10^−3^, and 1.0 × 10^−4^ s^−1^) were systematically examined in this work. Both normal and inverse PLC effects were discussed based on the experimental results. The feasibility of utilizing strain rate sensitivity as a crucial criterion for the occurrence of DSA or not was also explored in this work.

## 2. Experimental

The nominal composition of PM Co-Ni-based superalloy in this work is shown in Table 1. The alloy powders were prepared by argon atomization and then hot extruded into a rod. To homogenize alloy, solution treatment at 1200 °C for 3 h was conducted, followed by aging at 800 °C for 3 h, and finally cooling in air.

The H-shaped samples for the tensile tests were prepared by electron-discharge machining (EDM) with a tensile direction paralleling to the extruded direction. The length, thickness, and width of the gauge were set to 26.0, 2.0, and 3.4 mm, respectively. The tensile tests were conducted using a tensile testing machine (UTM 5105, China) at 200, 300, 400, 500, and 600 °C under various strain rates of 1.0 × 10^−2^, 1.0 × 10^−3^, and 1.0 × 10^−4^ s^−1^.

To probe the microstructure, the sheet samples were prepared by EDM, ground using grinding paper, and polished using an automatic lapping machine. The discs were subsequently etched in a mixed solution of 45% sulfuric acid (H_2_SO_4_), 43% nitric acid (HNO_3_), and 12% phosphoric acid (H_3_PO_4_) at room temperature at a voltage of 5 V for 15 s. Microstructural observation was performed on the samples after tensile tests by field emission scanning electron microscope (FESEM, Quanta 650 FEG, FEI) at 30 kV under the secondary electron image (SEI) mode. Electron backscattered diffraction (EBSD) was also used to measure the grain size and analyze texture characteristics using a step size of 0.5 μm.

## 3. Results

### 3.1. Initial Microstructures

**Figure 1:**
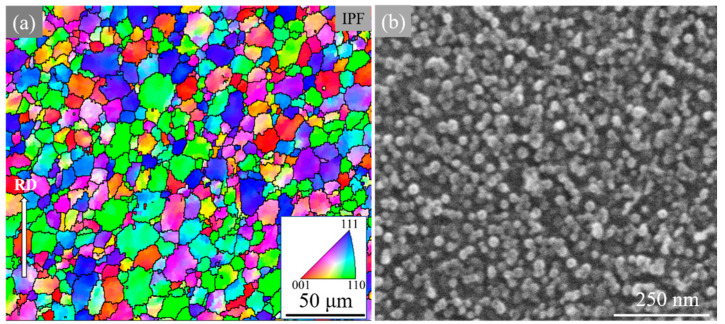
Microstructures of the Co-Ni-based superalloy: (**a**) EBSD result of alloy along the extrusion direction; (**b**) morphology of secondary γ′.

Figure 1a shows the EBSD result of the Co-Ni-based superalloy in terms of inverse pole figure (IPF) along the extrusion direction as indicated by the white arrow, and the average grain size is estimated to be around 7.7 μm. After etching, the morphology of γ′ was captured using FESEM and the result is depicted in Figure 1b. From it, only secondary γ′ phase precipitates, uniformly distributed in matrix with the average size of about 20 nm and volume fraction of near 30%, could be observed.

### 3.2. Serrated Flow Behavior and Characteristics

**Figure 2:**
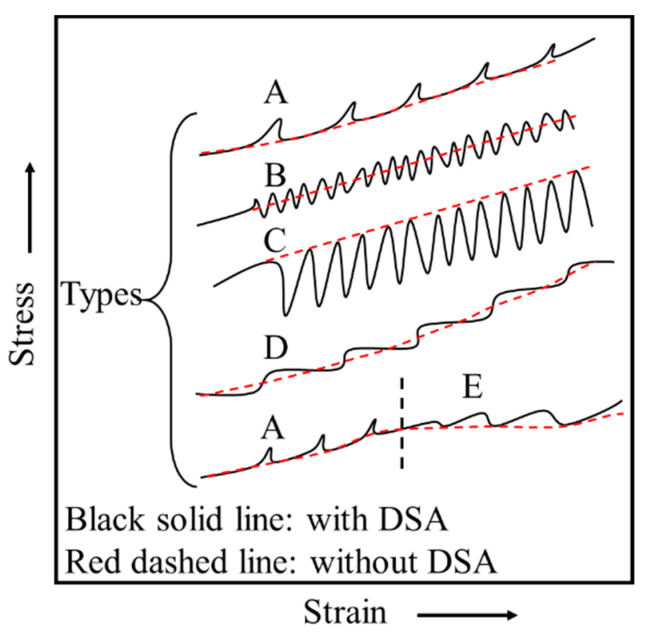
Schematic diagram of the classification of serrations types [11].

Figure 2 schematically shows five serration types (A-E) in stress–strain curves ascribed to the PLC effect. Type A is considered as locking serrations, characterized by a gradual rise followed by an abrupt drop to the general level of the stress–strain curve, and generally occurs in low temperature or high strain rate conditions. Type B is the oscillating along the general level of the stress–strain curve at a high frequency, usually developing from Type A or occurring at high temperature or low strain rate conditions. Type C occurs below the general level of the stress–strain curve and are therefore considered as unlocking serrations. Types D and E are similar to Type A, while there is little or no work hardening behavior. In this work, only types of A, B, and C were observed in the studied alloy.

**Figure 3:**
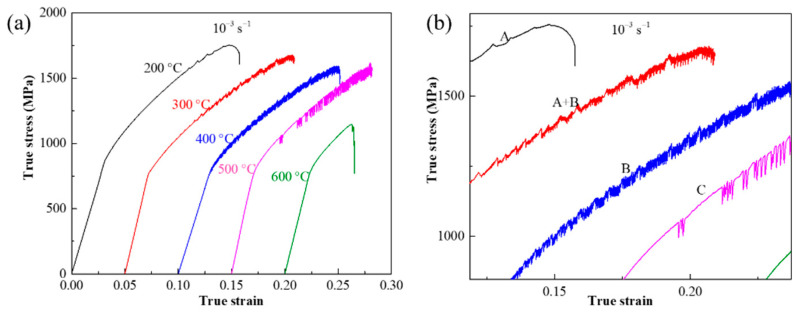
The true stress–strain curves of Co-Ni-based superalloy at the temperatures ranging from 200 to 600 °C with the strain rate of 1.0 × 10^−3^ s^−1^: (**a**) full view; (**b**) magnified views of (**a**).

**Figure 4:**
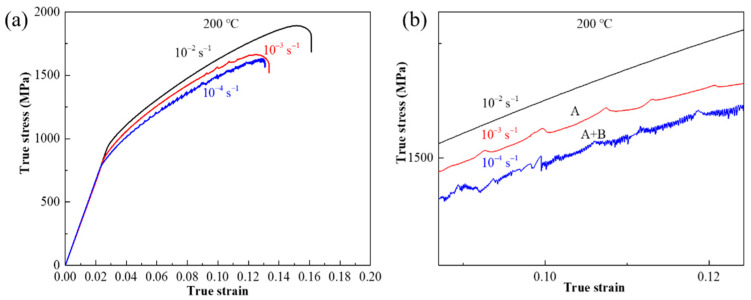
The true stress–strain curves of Co-Ni-based superalloy at the strain rate ranging from 1.0 × 10 ^−4^ s ^−1^ to 1.0 × 10 ^−2^ s ^−1^ at the temperature of 200 °C: (**a**) full view; (**b**) magnified views of (**a**).

Typical true stress–strain curves of Co-Ni-based superalloy at the temperatures ranging from 200 to 600 °C under the same strain rate of 1.0 × 10^−3^ s^−1^ are plotted in Figure 3a. It is obvious that serration flows appear at the temperatures below 600 °C. In addition, serration types can be clearly distinguished from the magnified picture given in Figure 3b. From it, as the temperature increases from 200 to 500 °C, the serration type transforms from A, A + B, B, to C. In terms of the influence of strain rate on the PLC effect under the condition of constant temperature. Typical true stress–strain curves of Co-Ni-based superalloy at the strain rate ranging from 1.0 × 10^−4^ to 1.0 × 10^−2^ s^−1^ and the constant temperature of 200 °C are shown in Figure 4. As the strain rate decreases to 1.0 × 10^−3^ s^−1^, the PLC effect phenomenon first appears; and the serration type changes from A to A + B, when strain rate further decreases to 1.0 × 10^−4^ s^−1^ (Figure 4b).

**Figure 5:**
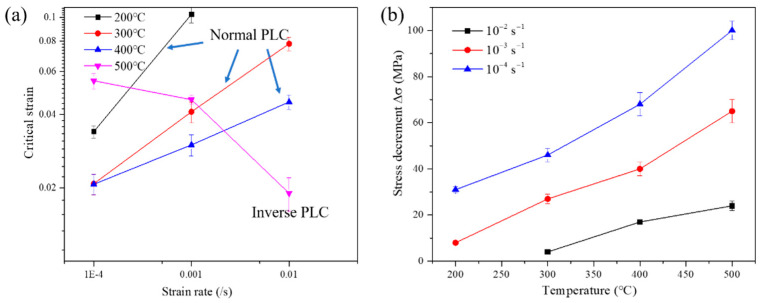
Relationships between: (**a**) critical strain, *ɛ*_c_, and strain rate, $\dot{\varepsilon }$; (**b**) stress decrement Δ*σ*, and temperatures, *T*.

The quantitative statistics regarding critical strain ($\varepsilon_{c}$, donated as the strain for the first serration to appear) and stress decrement (Δσ) evolving with strain rate and temperature during PLC regime are all summarized in Figure 5. In the range of 200 to 400 °C, under a constant strain rate of 1.0 × 10^−3^ s^−1^, increasing temperature decreases the $\varepsilon_{c}$ from 0.103 (200 °C) to 0.041 (300 °C), and then to 0.03 (400 °C). A opposite trend is observed as temperature further increases from 400 to 500 °C and the $\varepsilon_{c}$ increases from 0.03 to 0.046 (strain rate: 1.0 × 10^−3^ s^−1^). The former is called the normal PLC effect, in which $\varepsilon_{c}$ decreases with increasing temperature; the latter is termed the inverse PLC effect [22], whose $\varepsilon_{c}$ increases with increasing temperature (Figure 5a). It should be noted that, as temperature increases from 200 to 500 °C, the stress decrement Δσ is increasing for all strain rates, demonstrating the drag force from interaction between atoms and dislocation defects is enhanced with decreasing strain rate.

### 3.3. Mechanical Properties

**Figure 6:**
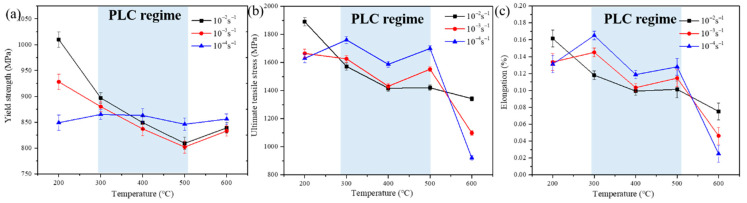
Effect of temperature and strain rate on: (**a**) yield strength, (**b**) ultimate tensile strength, and (**c**) elongation. The shaded area is the area where the PLC effect occurs.

Evolutions of yield strength ($\sigma_{Y}$), ultimate tensile strength ($\sigma_{U}$) and elongation ($e_{L}$) with increasing temperature under different strain rates $\dot{\varepsilon }$ are plotted in Figure 6. The shaded area is the area where the PLC effect occurs under all $\dot{\varepsilon }$. From Figure 6a, at a given temperature, $\sigma_{Y}$ increases as the $\dot{\varepsilon }$ changes from 1.0 × 10^−3^ to 1.0 × 10^−2^ s^−1^. Note that $\sigma_{Y}$ at the strain rate of 1.0 × 10^−4^ s^−1^ shows a weak temperature dependence, thus leading to high $\sigma_{Y}$ under high temperatures. As for $\sigma_{U}$, it decreases with increasing temperature *T* at all strain rates. An opposite relationship between $\sigma_{U}$ and $\dot{\varepsilon }$ in the area with or without the PLC effect is observed. $\sigma_{U}$ increases with decreasing $\dot{\varepsilon }$ in the PLC effect regime while increases with increasing $\dot{\varepsilon }$ in the area without the PLC effect. Variation of $e_{L}$ with T at various $\dot{\varepsilon }$ is plotted in Figure 6c. At a given temperature, in the PLC effect area, $e_{L}$ increases with decreasing $\dot{\varepsilon .}$ In areas without the PLC effect, $e_{L}$ increases with increasing $\dot{\varepsilon .}$ Contrary to the existing experimental results [14,17,20,21], the tensile strength and elongation of the alloy in the PLC effect regime of this alloy increase roughly as the $\dot{\varepsilon }$ decreases. The underlying mechanism is explored in the following section.

## 4. Discussion

It is well accepted that the PLC effect arises from the interactions between the solute atoms and mobile dislocations [23]. The necessary condition for the occurrence of the PLC effect is that the aging time of the solute $t_{a}$ is less than the dislocation breakaway time $t_{w}$ ($t_{a}$ ≤ $t_{w}$) [5]. Consistent with the existing results [8,17,21], increasing the temperature and decreasing the strain rate can promote the occurrence of the PLC effect. Based on the existing experimental results, the activation energy of the PLC effect and the increased ductility are discussed, and the feasibility of the SRS criterion and the normal and inverse PLC effects are analyzed.

### 4.1. Activation Energy

**Figure 7:**
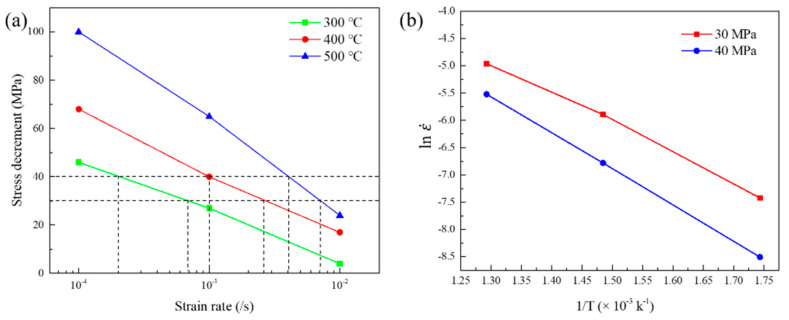
Plots for determination of activation energy: (**a**) Variation of stress decrement, Δ*σ*, with strain rate, $\dot{\varepsilon }$, at different temperatures, *T*. (**b**) ln $\dot{\varepsilon }$ vs. 1/*T* using intercepts from (**a**).

Activation energy calculation was conducted on the present alloy and compared with the solute migration energy to figure out which atoms should be responsible for the serrated flow in the present alloy. The calculation method is adapted from the work studied by Hayes et al. [24,25]:

(1) $$Q=-R{\left[\frac{\Delta \ln\dot{\varepsilon }}{\Delta \frac{1}{T}}\right]}_{\Delta \sigma ,\varepsilon }$$

where Q is the activation energy for serrated flow and *R* is the gas constant. In Figure 7a, stress decrement $\Delta_{\sigma }$ of 30 and 40 MPa at 300, 400, and 500 °C is picked, then the corresponding strain rate $\dot{\varepsilon }$ can be obtained. Taking $\Delta_{\sigma }$ and $\dot{\varepsilon }$ into plot $\ln\dot{\varepsilon }$ vs. $\frac{1}{T}$ as shown in Figure 7b. In the normal PLC regime (300 and 400 °C), 125 and 141 kJ/mol are obtained using $\Delta_{\sigma }$ values of 30 and 40 MPa, take the average value 131 kJ/mol as the activation energy in present alloy. For the inverse PLC regime (400 and 500 °C), the average value of 139 kJ/mol is calculated. Similar activation energy data indicate the same dominant element in normal and inverse PLC regime.

Before discussing the activation energy of the solute, we must first determine how the solute diffuses. Based on previous study [14], due to the lower diffusion energy barrier, pipe diffusion has priority over bulk diffusion, and recently Garbrecht et al. [26] directly observed the diffusion of solute atoms along dislocations. The activation energies for dislocation pipe diffusion can be calculated as $Q_{\mathrm{pipe}}$ = 0.65 × $Q_{\mathrm{bulk}}$. It has been reported that the activation energy for bulk diffusion of Mo in Ni is 213 kJ/mol. Accordingly, the solute migration energy for pipe diffusion of Mo can be estimated as 139 kJ/mol, which is comparable to activation energy (133 kJ/mol) for serrated flow in the present alloy. Herein, the substitutional atom Mo is considered to take primary responsibility for the PLC effect in present alloy.

### 4.2. Interpretation Abnormal Mechanical Properties in PLC Effect Regime

As mentioned above, the temperature at which the PLC effect occurs is close to the service temperature of the superalloy. We are concerned about whether the PLC effect has an adverse effect on mechanical properties. Since the PLC effect can be promoted by increasing temperature T and decreasing strain rate$\;\dot{\varepsilon }$, we will explain the relevance of mechanical properties from these two perspectives. As plotted in Figure 6a–c, variation of yield strength ($\sigma_{Y}$), ultimate tensile strength ($\sigma_{U}$), and elongation ($e_{L}$) with T at various $\dot{\varepsilon }$ can be observed. In the area where the PLC effect completely occurs: (1) the $\sigma_{Y}$, $\sigma_{U}$, as well as $e_{L}$ decrease as the T rises, but not by much; (2) the $\sigma_{Y}$, $\sigma_{U}$, as well as $e_{L}$ increase as the $\dot{\varepsilon }$ decreases. These results are contrary to the work by Pu et al. [14], where the $\sigma_{U}$ decreases drastically with the T, and the $e_{L}$ decreases with the decrease in the $\dot{\varepsilon }$; and differ from the work by Tian et al. [21], where $\sigma_{Y}$, $\sigma_{U}$ as well as $e_{L}$ do not vary much with T and $\dot{\varepsilon }$. Stable mechanical properties in the PLC effect range ensure the safety of the alloy.

### 4.3. Interpretation of SRS and Normal/Inverse PLC Effect

#### 4.3.1. Interpretation of Positive and Negative SRS

**Figure 8:**
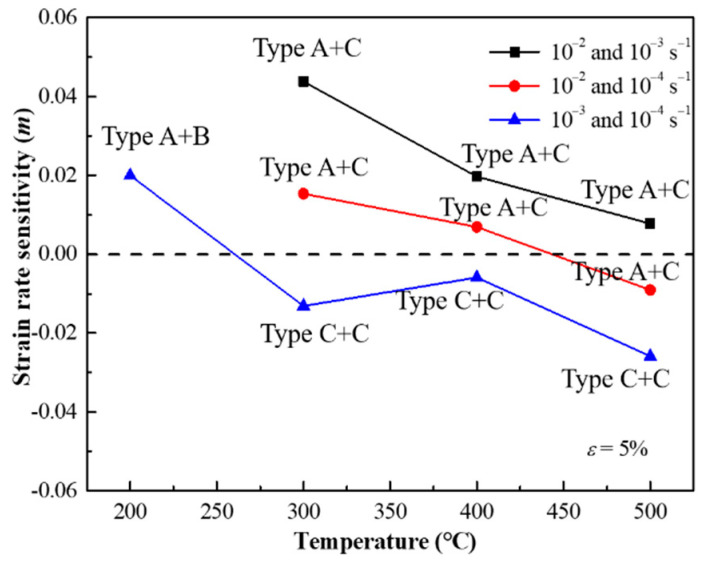
Variation of strain rate sensitivity (*m*) between different strain rate ($\dot{\varepsilon }$) vs. temperature (*T*) at a true plastic strain, *ɛ*, of 5%.

Serrations are the manifestation of macroscopic scale, which cannot be used to predict whether the PLC effect happens. It has been accepted that the indicator of the PLC effect is the negative strain rate sensitivity (SRS), which was first summarized from the experiment by Penning et al. [27]. Later, it was confirmed by many researchers [13,25,28]. the strain rate sensitivity exponent (*m*) at a given temperature (T) and certain strain (ε) was calculated using stress–strain data from the tensile tests by the following Equation (2) [17]:

(2) $$m={\frac{\log\left(\sigma_{1}/\sigma_{2}\right)}{\log\left({\dot{\varepsilon }}_{1}/{\dot{\varepsilon }}_{2}\right)}|}_{\varepsilon ,T}$$

where $\sigma_{1}$ and $\sigma_{2}$ are the flow stresses at strain rates ${\dot{\varepsilon }}_{1}$ and ${\dot{\varepsilon }}_{2}$, respectively. Values of (*m*) at certain strain (ε=5%) were calculated with different strain rates, respectively, as shown in Figure 8, attached the PLC effect type of the serration of each data point. Both positive and negative *m* are observed in the PLC effect regime. It is interested to find that positive *m* comes from different serration types (A with B/C) while negative *m* mostly derived from same serration types (C with C).

**Figure 9:**
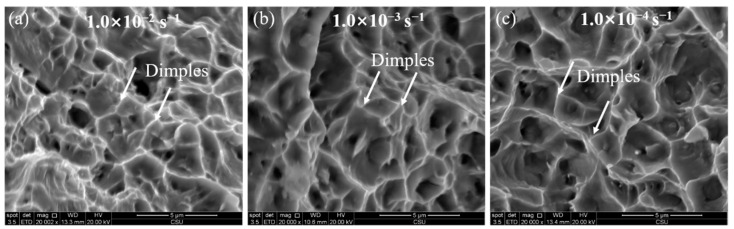
Fracture characterization at 300 °C under different strain rates: (**a**) 1.0 × 10 ^−2^ s ^−1^, (**b**) 1.0 × 10 ^−3^ s ^−1^ and (**c**) 1.0 × 10^−4^ s ^−1^.

As can be observed in most alloys, positive *m* brings a strength increasing with the increasing strain rate [29]. A more rapid deformation then results in a higher stress, which cause more uniform deformation. The effect of negative *m* is to allow strain localization [30] and thus inhomogeneous yielding to occur. Therefore, low elongation is expected [14]. However, an increasing elongation with decreasing strain rate is observed in this study as shown in Figure 6c. The fracture shown in Figure 9 also supports this view, as the strain rate decreases, larger dimples are observed. A similar work carried out by Tian et al. [21] in a Ni-Co-based superalloy obtained the same result, there are many twins in the structure after stretching. The greatest difference between Co-Ni-based alloys and traditional Ni-based superalloys is its low stacking fault energy [31]. When the dislocation movement is limited, twins can be served as a cooperative method to coordinate the deformation [32]. In the work of the casting state alloy with the same composition [3], a large number of twins are observed after deformation, which could explain the increase in plasticity in this study.

In general, the negative *m* as a criterion for the occurrence of the PLC effect is accepted by most scientists. There is indeed a positive *m* in the PLC effect regime in this work. Therefore, we add a restriction condition used by the criterion, that is, the same type of serrations, the criterion of negative *m* still valid.

#### 4.3.2. Interpretation of Normal and Inverse PLC Effect

It is well accepted that increasing temperature and decreasing strain rate can play a positive role in the PLC effect. Unfortunately, only suitable from room temperature to 400 °C, in other words, the normal PLC effect. There is a lack of solid interpretation for the inverse PLC effect (400 to 500 °C). We are here to make some explanations based on the experimental results and previous works.

**Figure 10:**
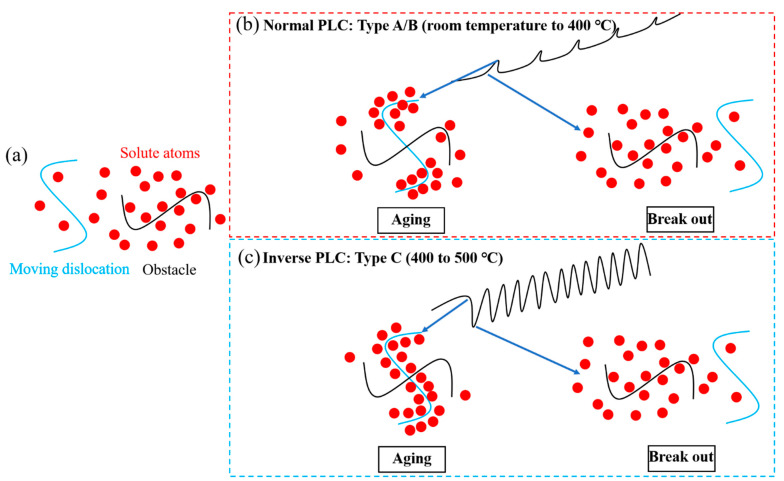
Schematic for the interactions between mobile dislocations and solutes at different deformation conditions: (**a**) Conditions before locking/unlocking; (**b**) Normal PLC: Type A/B (room temperature to 400 °C); (**c**) Inverse PLC: Type C (400 to 500 °C).

In our opinion, the difference between the normal and inverse PLC effects is whether locking or unlocking dominates. The supporting views are as follows:

1. Serrations type A and B occurs in normal PLC while type C emerge in inverse PLC [11,12]. Type A and B are locking serrations, the pinning force brought by the solute atoms is reflected in the part above the normal stress–strain curve. Type C is unlocking serrations, the serration part is completely below the normal stress–strain curve. The hypothesis is supported by the work of Fu [33] in an Al-Mg alloy 5456, who proposed that for the normal behavior, the critical strain depends on the first pinning; for the inverse behavior, the critical strain depends on the first unpinning.
2. Serrations usually occur after a certain strain,$\;\varepsilon_{c}$, the preparatory period of the PLC effect, the magnitude of which reflects the difficulty of the PLC effect’s occurrence. In the normal PLC effect, $\varepsilon_{c}$ decreases with increasing temperature T, while it increases in the inverse PLC effect. These contradictory results can be illuminated with the schematic diagram in Figure 10. In the condition of the normal PLC effect shown in Figure 10b, part of the solute atoms can pin the dislocation, or part of the dislocation is pinned by the solute atoms. Increasing T shortens $\varepsilon_{c}$ by accelerating the diffusion of atoms and increasing applied stress by strengthens the interaction between dislocations and atoms, which can be observed from stress decrement (Δ*σ*). At the transition temperature from normal to inverse, $\varepsilon_{c}$ is close to 0 (the PLC effect happens when it enters the plastic deformation). In the inverse PLC effect under higher T shown in Figure 10c, motivated by thermal activation, almost all solutes and all parts of dislocations are involved in pinning, $\varepsilon_{c}$ extends by increasing T as is not easy for dislocations to escape from solute atoms. A lager stress (strain) is required to break them. In the $\varepsilon_{c}$, the first serration appears with a stress drop. As the temperature further increases, the PLC effect disappears because the dislocations cannot escape the thermally activated solute atomic atmosphere.

## 5. Conclusions

The Portevin-Le Châtelier (PLC) effect in a powder metallurgy (PM) Co–Ni-based superalloy was systematic examined. The mechanisms of the PLC effect were analyzed by the characterization of serrations. The main conclusions are as follows:

1. The average activation energy for serrations is 133 kJ/mol, which is comparable with migration energy of Mo (139 kJ/mol) through pipe diffusion. Accordingly, we infer that Mo atoms should be responsible for the PLC effect in this alloy.
2. In the PLC effect regime, yield strength, ultimate tensile strength, as well as elongation show limited drop with temperature. Ultimate tensile strength and elongation increase with decreasing strain rate at a given temperature, which ensure the stability of the alloy in the PLC effect area.
3. Both negative and positive strain rate sensitivity are shown in the PLC effect regime: the former is derived from different serration types while the latter is generally the same serration type.
4. The normal PLC effect is observed at room temperature to 400 °C while the inverse PLC effect is shown at temperatures 400 to 500 °C. The critical strain in the normal PLC effect represents a stronger interaction between solute atoms and dislocations, and the one in inverse represents the first break between dislocation and solute atoms.

## Figures and Tables

**Table 1:** The nominal chemical composition of studied Co-Ni-based alloy (wt.%).

	Co	Ni	Cr	Mo	Nb	Al	Ti	Fe	C
Composition (wt.%)	Bal.	36.0	17.5	8.0	3.0	2.0	0.8	1.6	0.15
